# Systematic Review and Meta-Analysis on Human African Trypanocide Resistance

**DOI:** 10.3390/pathogens11101100

**Published:** 2022-09-25

**Authors:** Keneth Iceland Kasozi, Ewan Thomas MacLeod, Susan Christina Welburn

**Affiliations:** 1 Infection Medicine, Deanery of Biomedical Sciences, Edinburgh Medical School, College of Medicine and Veterinary Medicine, The University of Edinburgh, Edinburgh EH8 9JZ, UK; 2 School of Medicine, Kabale University, Kabale P.O. Box 317, Uganda; 3 Zhejiang University-University of Edinburgh Joint Institute, Zhejiang University, International Campus, 718 East Haizhou Road, Haining 314400, China

**Keywords:** human African trypanosomiasis, trypanosomes, drug resistance, pentamidines, nifurtimox/eflornithine combination therapy, fexinidazole, NECT, *Tb*AT1, amino-aquapurine transporters, amino acid transporters, *trypanosoma brucei rhodesiense*, *trypanosoma brucei gambiense*, neglected tropical diseases

## Abstract

Background Human African trypanocide resistance (HATr) is a challenge for the eradication of Human African Trypansomiaisis (HAT) following the widespread emergence of increased monotherapy drug treatment failures against *Trypanosoma brucei* *gambiense* and *T. b. rhodesiense* that are associated with changes in pathogen receptors. Methods: Electronic searches of 12 databases and 3 Google search websites for human African trypanocide resistance were performed using a keyword search criterion applied to both laboratory and clinical studies. Fifty-one publications were identified and included in this study using the PRISMA checklist. Data were analyzed using RevMan and random effect sizes were computed for the statistics at the 95% confidence interval. Results: Pentamidine/melarsoprol/nifurtimox cross-resistance is associated with loss of the *T. brucei* adenosine transporter 1/purine 2 gene (*Tb*AT1/P2), aquaglyceroporins (*Tb*AQP) 2 and 3, followed by the high affinity pentamidine melarsoprol transporter (HAPT) 1. In addition, the loss of the amino acid transporter (AAT) 6 is associated with eflornithine resistance. Nifurtimox/eflornithine combination therapy resistance is associated with AAT6 and nitroreductase loss, and high resistance and parasite regrowth is responsible for treatment relapse. In clinical studies, the *Tb*AT1 proportion of total random effects was 68% (95% CI: 38.0–91.6); I^2^ = 96.99% (95% CI: 94.6–98.3). Treatment failure rates were highest with melarsoprol followed by eflornithine at 41.49% (95% CI: 24.94–59.09) and 6.56% (3.06–11.25) respectively. HATr-resistant phenotypes used in most laboratory experiments demonstrated significantly higher pentamidine resistance than other trypanocides. Conclusion: The emergence of drug resistance across the spectrum of trypanocidal agents that are used to treat HAT is a major threat to the global WHO target to eliminate HAT by 2030. *T. brucei* strains were largely resistant to diamidines and the use of high trypanocide concentrations in clinical studies have proved fatal in humans. Studies to develop novel chemotherapeutical agents and identify alternative protein targets could help to reduce the emergence and spread of HATr.

## 1. Introduction

The World Health Organization (WHO) has set 2030 as a target for the elimination of human African trypanosomiasis (HAT) [1]; however, the development of drug-resistant phenotypes (see [2] on trypanocide resistance) in resource-poor countries affected by HAT presents a major challenge for HAT control. In Africa, HAT is caused by *Trypanosoma brucei gambiense* (TBG, chronic variant) and *T. b. rhodesiense* (TBR, acute variant), also referred to as gHAT and rHAT, respectively. Uganda has the misfortune to harbor both sub-species within its borders [3]. Trypanosomes are able to evade host immune defenses through a process of antigenic variation (while each genome contains over 10^3^ distinct variable surface glycoprotein (VSG) genes, every trypanosome typically expresses a single VSG that rapidly switches [4], compromising any efforts to develop vaccines).

Globally, six major drugs are available for treatment of HAT depending on the stage of the infection: pentamidine, suramin, melarsoprol, eflornithine, nifurtimox/eflornithine combination therapy (NECT), and fexinidazole (see [2] on HAT pharmaceutics and limitations of current approved therapies). The emergence of human African trypanocide resistance (HATr) has undermined the use of monotherapy for HAT treatment.

Pentamidine was the first antiprotozoal diamidine to be routinely used for HAT treatment, its mode of action serving to disrupt the AATT-rich portions of *Trypanosoma* DNA and suppress mitochondrial activity [5]. Pentamidine resistance has been linked to changes in the transmembrane transport of the drug i.e., *Trypanosoma brucei* adenosine transporter 1/purine 2 (*Tb*AT1/P2) and high-affinity pentamidine transporter 1 (HAPT1) [5,6,7]. Melarsoprol is an arsenical with which resistance has been associated with aquaglyceroporin transporters 2/3 (AQP2/3) in trypanosomes [8]. Resistance has led to the adoption of combination nifurtimox/eflornithine combination therapy (NECT), which has low toxicity and a shortened therapeutic period, although weekly intravenous infusions have proved challenging in resource-limited settings [9]. In early stages, pentamidine (TBG) and suramin (TBR) are used while melarsoprol (TBG and TBR) and eflornithine (TBG) are recommended for late-stage HAT [3]. New drugs, including pafuramidine maleate for early HAT, have failed, while acoziborole for stage 1 and 2 HAT is under continuous review [10], demonstrating the need to develop novel therapies; for example, fexinidazole is easier to administer since it is an oral medication [11,12]. The objective of the current study was to identify major parasitic markers in HATr associated with clinical studies (field surveys and clinical trials) and experimental (laboratory-based) studies.

## 2. Methods

### 2.1. Study Design

Multiple electronic databases were searched using the Ovid interface: AMED (Allied and Complementary Medicine) 1985 to March 2022, CAB Abstracts 1973 to 2022 Week 13, APA PsycInfo 1806 to March Week 4 2022, Books@Ovid 28 March 2022, Journals@Ovid Full Text 01 April 2022, Your Journals@Ovid, APA PsycArticles Full Text, CAB Abstracts 1910 to 1989, Embase Classic+Embase 1947 to 01 April 2022, Global Health 1910 to 2022 Week 13, Ovid MEDLINE(R), and Epub Ahead of Print, In-Process, In-Data-Review & Other Non-Indexed Citations, Daily and Versions 1946 to 01 April 2022. These searches generated a total of 172 publications and the Web of Science generated 111 publications using the following keywords as shown in Supplementary File S1.

ALL = ((african trypanosomiasis)) OR ALL = (trypanosoma brucei)) OR ALL = (‘tsetse fly-borne diseases’ OR ‘HAT’ or ‘human African trypanosomiasis’))) AND ALL = ((trypanosoma brucei gambiense) or (trypanosoma brucei rhodesiense))) AND ALL = ((suramin OR melarsoprol OR eflornithine OR nifurtimox OR pentamidine OR (NECT or (Nifurtimox Eflornithine Combination Therapy))))) AND ALL = (trypanocides resistance or drug resistance).

**Figure 1:**
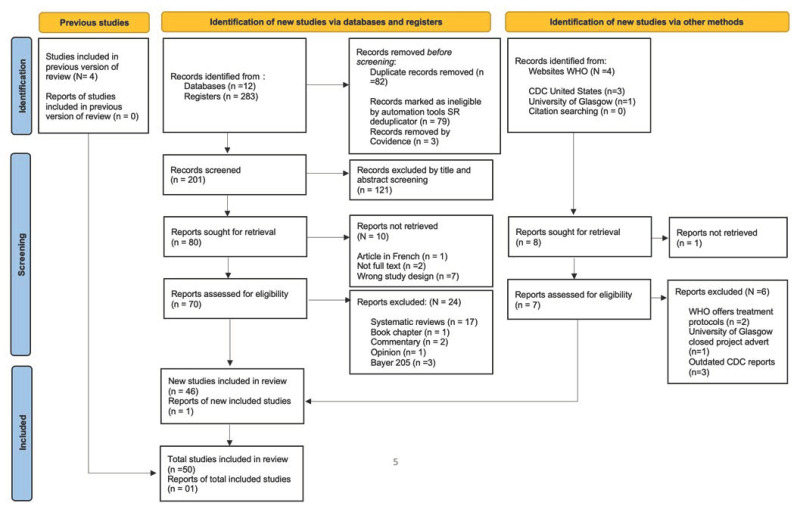
PRISMA checklist showing studies from database, Google search, and citation review.

Grey literature was searched on Google using the WHO and CDC websites, and citations in systematic reviews were also searched for papers that might have been missed (Figure 1).

### 2.2. Article Screening on Inclusion and Exclusion

Data files were exported to EndNote 2020 and all papers were merged (N = 283). The SR depublicator removed 79 duplicates. A total of 204 papers were then exported to Covidence, which removed 3 additional duplicates, and these were confirmed by authors. Title and abstract screening removed 121 papers and only 80 papers were subjected to full-text review. After removing review articles, a total of 46 papers were acquired from the search and an additional 5 papers were added (i.e., 1 from Google search and 4 from review searches), as shown in Figure 1.

### 2.3. Statistical Analysis

Data were exported into MS Excel and categorized into laboratory studies (cell culture and rodents) and clinical studies (involving humans). These data were descriptively presented in tables while quantitative data were analyzed using RevMan^®^ for meta-analysis using proportions and random effect sizes at 95% confidence interval. Data on the impact of resistance were analyzed using GraphPad Prism version 6 and posthoc Tukey’s tests were conducted and represented with different superscripts (a,b) being used to indicate significant differences (*p* < 0.05)

## 3. Results and Discussion

### 3.1. Description of Human African Trypanocide Resistance in the Study

Pentamidine/melarsoprol cross-resistance (PMXR) is associated with loss of and mutations in *Tb*AT1/P2 genes [13], followed by mitochondrial, post translational activator XAC1, and flagellar genes [14]. The loss of *Tb*AT1 alone does not effectively prevent endocytosis of pentamidine and melarsoprol (see [15], melarsoprol has other entry targets); however, a loss of aquaglyceroprotein (*Tb*AQP, see [16], AQPs are important for viability and osmoregulation) has been associated with complete loss of trypanocide endocytosis [17]. In particular, the loss of *Tb*AQP2/3 following point mutations in *Tb*AT1 leads to a loss of the high-affinity pentamidine/melarsoprol transporter (HAPMT) [18,19]. In pentamidine diamidine resistance, HAPT1 and low-affinity pentamidine transporter 1 (LAPT1) are responsible for the residual uptake of melaninophenyl arsenic [20]. The presence of multiple resistance genes in single parasites contributes to a markedly resistant phenotype. For example, *Tb*AT1 and *T. brucei* multidrug-resistant pentamidine-associated gene (*Tb*MRPA) further complicate melarsoprol resistance [21] (Table 1).

These findings are important since pentamidine and melarsoprol have been the major trypanocides involved in HAT chemotherapy for over 60 years [22]. Cross-resistance between melarsoprol-pentamidine, diminazene aceturate, isometamidium chloride has been reported [23]. Although drugs used in HAT are different from those used for animal African trypanosomiasis, pentamidines/diamidines with similar pathogen targets (*Tb*AT1/P2) contribute to this selection pressure [24]. This is important since TBR and TBG have been isolated from livestock species that have been recognized as maintenance hosts (see [25] in pigs, [26] in small ruminants, and [27] in cattle) and sources of re-infection and introduction of resistant phenotypes in humans [28].

Nifurtimox is metabolized rapidly and its metabolites are not effective against *T. brucei* s.l., thus reducing its therapeutical effect [29]. Similar results have been reported for *Tb*AT1, where the loss of P2 favors trypanosome survival [30]. The loss of amino acid transporter (AAT) 6 leads to increased eflornithine resistance [24,25] while NECT resistance is associated with multiple loss of function of AAT6 and nitroreductase (NTR) [26,27]. Resistance to arsenicals and diamidine is associated with HAPT loss [31] while *Tb*AT1 loss disrupts the uptake of diamidines [32]. In addition, cross-resistance in arsenical and suramin [33], arsenical/melarsoprol/pentamidine and diminazene aceturate [15] as well as isometamidium and diminazene aceturate, which are mainly used in livestock [34], raises major public health concerns since pentamidines and diminazene aceturate are used in both humans and animals [24]. Furthermore, the presence of putative nascent polypeptide associated complex (NAC) isoforms [35] presents a dilemma with opportunities for drug targets and challenges to address HATr (Table 1). Nifurtimox resistance has also been associated with *Tb*AT1/P2 (contrary to previous assumptions [36] in which no relationship was made); however, AAT6 loss has been associated with eflornithine resistance. In NECT resistance, multiple loss of AAT6 and NTR is the hallmark of drug resistance. Nifurtimox–fexinidazole resistance is associated with the rapid metabolism of the sulfoxide and sulfone forms of this compound [22]. Since nifurtimox was first developed for use against American trypanosomiasis [37], it is apparent that further research exploring combination therapy could yield more efficient trypanocides.

Resistance to suramin (developed in 1916) has been associated with switching of VSG expression [31,38]. Furthermore, other mechanisms associated with ATP production, metabolism, cell cycle, and genome segregation [39] will continue to offer opportunities for continued research (Table 1). Although the cellular pathways involved in suramin resistance remain to be discovered (see [40] where resistance was postulated to develop after changes in the drug target by expression of drug extrusion mechanisms), since each parasite contains over 1000 VSG genes, expression switching to one particular VSGSur implies that it is close to impossible to eliminate suramin resistance in a population [41].

**Table 1:** Characterization of pathogens, interventions used, and gene targets in human African trypanosome resistance in laboratory studies.

Study	Ref	Study Population	Source of Pathogen	Intervention/Drugs Used	Gene Targets for Resistance
Bernhard 2007	[13]	Mice	TBR	Pentamidine-melarsoprol	*Tb*AT1 loss indicated cross-resistance on both compounds
Carter 2020	[14]	*T brucei* ORFeome	Parasite library	Melarsoprol	Genes encoding trypanothione Mitochondrial and flagellar gene expression (post translational activator XAC1).
Scott 1997	[15]	Procyclics	TBB	Cross resistance with arsenical-melarsoprol-pentamidine and diminazene aceturate	Melarsoprol can enter parasite through another route than *Tb*AT1
Jeacock 2017	[16]	Mice	TBB/TBG	AQP2 disrupts glycerol transport	AQPs important for viability and osmoregulation
Graf 2013	[17]	Procyclics	TBR and TBG isolates from 7 African countries	Pentamidine and melarsoprol	*Tb*AT1 loss leads to a loss of transporter activity Aquaglyceroprotein (*Tb*AQP2)
Graf 2016	[18]	Procyclics	TBR from male patient in Tanzania	Pentamidine and melarsoprol	Loss of transporter genes *Tb*AQP2/3 Point mutation renders *Tb*AT1 useless (lacks HAPMT = high affinity pentamidine-melarsoprol transporter)
Graf 2015	[19]	Procyclics	TBG	Pentamidine and melarsoprol	*Tb*AQP2 reintroduced reversed resistance
Matovu 2003	[20]	Procyclics/mice	TBB	Loss of *Tb*AT1/P2 in pentamidine and diamidine uptake	HAPT1 and LAPT1 responsible for residual uptake of melaminophenyl arsenical
Lusher 2006	[21]	Procyclics	TBB	Melarsoprol resistance	*Tb*AT1 and *Tb*MRPA when both present lead to significant decrease in drug influx
Sokolova 2010	[29]	Procyclics	TB strain *	Nifurtimox-resistant cell lines	Nifurtimox metabolized fast and metabolites not effective on pathogen
Geiser 2005	[30]	Procyclics	TBB strain BS 221	Adenosine metabolites	P1/P2 *Tb*AT1 loss. Conditions other than drugs themselves may favor loss of P1 to increase pathogen survival in bloodstream form of the parasite.
Burkard 2011	[42]	RNAi induction	RNAi library	NA	Loss of *Tb*AT1 leads to melarsoprol resistance. Loss of AAT6 leads to increased eflornithine resistance.
Vincent 2010	[43]	Procyclics	*T. brucei* strain 427 wildtype	Eflornithine resistance	Ornithine decarboxylase unaltered in parasite. Deletion of *Tb*AAT6
Baker 2011	[44]	Procyclics	TBR	NECT	Loss of amino acid transporter (AAT6) and nitroreductase (NTR) induces resistance
Wyllie 2016	[38]	Procyclic/mice	Non-specific trypanosome used	Nifurtimox	NTR resistance determinants
Bridges 2007	[31]	Rats	TBG	Arsenical and diamidine	High-affinity pentamidine transporter (HAPT) loss for cross-resistance
Lanteri 2006	[32]	Procyclics/mice	TBB	2,5-BIS(4-amidinophenyl) furan (DB75) (diamidine)	Loss of *Tb*AT1 leads to loss in uptake of DB75.
Scott 1996	[33]	Mice	TBB from Tanzania TBG from man in Ivory cost	Cross-resistance to MelCy and suramin	Differences in in vivo and in vitro results indicated alteration in surface adenosine transporters.
Matovu 1997	[34]	Humans and livestock	TBR in Uganda	Resistant to ISM, DA	Cross-species resistance
Foucher 2006	[35]	Procyclics	TBG clones	Cymelarsan	Putative NAC isoform loss. Alterations in the activity of the enzyme that generates protein translation modifiers.
Wiedemar 2019	[45]	Procyclics	TBB	VSG expression has impact on suramin sensitivity and uptake	Decrease specific receptor-mediated endocytosis
Zeelen 2021	[41]	Procyclics	TBR	VSG-suramin binding interactions	Resistance phenotype dependent on suramin binding with VSG^sur^
Worthen 2010	[39]	Mice	Modeling HATr resistance	Pentamidine, prostaglandin D2, quercetin, etoposide, camptothecin, tetrahydroquinoline	Defects in mitochondrial activity, ROS, cell cycle, and genome segregation.
Bacchi 1994	[46]	Mice	TBR from Kenya	Combination of DFMO, eflornithine, and ornidyl	Cure rate in days
Bacchi 1993	[47]	Mice	TBR from Kenya	DFMO resistance	S-adenosylmethionine metabolism increases resistance
Pati 2014	[48]	Humans	TBG in DRC	Melarsoprol	Relapse following mutations in AQP2/3
Matovu 2001	[49]	Procyclics	TBG from northwestern Uganda	Melarsoprol	Elevated MIC
Brun 2001	[50]	Humans and then mice	TBR KETRI and EATRO trypanosome isolates from Kenya STIB 241 and STIB 704 from Uganda	Melarsoprol	Cure rate
Hawking 1941	[51]	Mice inoculated with patient blood/CSF	TBR	Tryparsamide	Relapse
Kagira 2007	[52]	Mice	TBR in patients from Uganda and Kenya	Melarsoprol-pentamidine cross resistance (MPXR)	Relapse
Kibona 2006	[53]	Mice	TBR from Tanzania	Melarsoprol resistance DA resistance at 14 mg/kg	High minimum inhibition concentrations (MIC) and IC50
Maina 2007	[54]	Humans/mice	TBG in South Sudan	Melarsoprol resistance	*TB*AT1/P2 loss
Mpia 2002	[55]	Humans	TBG	Combination of eflornithine and melarsoprol	Cleared infection though toxicity concerns raised.
Munday 2014	[56]	Procyclics	*TB*B	Pentamidine, melaminophenyl arsenic (PA)	*Tb*AQP2 is HAPAT and source of resistance
Munday 2015	[57]	Procyclics	*TB*B	*Tb*AT1	Residues F19, D140, and F316 interact with the *Tb*AT1 substrate.
Mutuku 2021	[58]	Mice	TBR in Busoga, Uganda	Suramin resistance	Differential pathogenicity in TBR strains
Nerima 2007	[59]	Mice	TBG northwest Uganda	Detection of mutant P2/*Tb*AT1	Allele-specific PCR is cheaper than *Sfa*N1 RFLP for screening of *Tb*AT1
Nnadi 2019	[60]	Procyclics	*T. congo* *TB*B	Holarrhetine	*Tb*AT1, AQP1-3
Sanderson 2009	[61]	Mice	*TB*B	Pentamidine	Blood–brain barrier via P-glycoprotein and multiple drug resistance-associated protein transporters.

Key: Superscript (*) = strain genus not defined in the article. NA = not applicable.

### 3.2. Human African Trypanocide Resistance in Clinical Studies

In clinical studies, relapse/cure rates have been used to study HATr (see [46] on using DL-α-difluoromethylornithine (DFMO, also referred to as eflornithine in most studies) with suramin against arsenical refractory HAT in mice). This is important since DFMO alone has been associated with resistance [47]. Combination of metronidazole and suramin were used to address arsenical resistance in HATr in Zambia [62], while high doses of nifurtimox have also been explored in the Democratic Republic of the Congo (DRC) [63,64]. Melarsoprol resistance in DRC has been associated with mutations in the AQP2/3 gene [48] and parasite regrowth [65]. Human samples from Tanzania and Ivory Coast showed cross-resistance to melarsoprol and suramin due to alterations in *Tb*AT1 [33] or other transport mechanisms [15] (Table 2).

Melarsoprol resistance in Uganda and Tanzania has also been associated with mutations in the *Tb*AT1 gene [66], and high minimum inhibition concentration (MIC) titers (see [49]) in Uganda). In DRC, melarsoprol alone is no longer used in late HAT [67] and eflornithine is now used at this stage [68]. The increasing levels of HATr has led to the promotion of combination therapy [69], as well as capital to invest in the discovery of more potent drugs better than the current drugs on the market.

**Table 2:** Human African trypanosome resistance in human populations with location and drugs used during interventions.

Study	Ref	Study Population	Source of Pathogen	Intervention/Drugs Used	Marker for Resistance
Foulkes 1996	[62]	Human	TBR in Zambia	Melarsoprol resistance Then given suramin	Melarsoprol refractory period/relapse
Pepin 1989	[63]	Human	TBG in DRC	Nifurtimox for arseno-resistance	No relapse
Pepin 1992	[64]	Human	TBG in DRC	Arsenic resistance	High-dose nifurtimox
Richardson 2016	[65]	Human	TBG in DRC		Parasite regrowth leads to relapse not reinfection
Matovu 2001	[66]	Human	TBR in Uganda TBG from Angola	Melarsoprol	Mutated *Tb*AT1
Burri 2001	[70]	Human	TBG in M’banza Congo, Angola	Melarsoprol	Cure rate in patients
Kazibwe 2009	[71]	Human	TBG from northwestern Uganda	Melarsoprol withdrawal	*Tb*AT1/P2 present in pathogen
Pyana 2015	[72]	Human	TBG in DRC	Pentamidine melarsoprol resistance	Cure depends on patient factors such as nutrition, immunological and coinfections with other pathogens
Balasegaram 2006	[73]	Human	TBG in DRC	Pentamidine	Relapse rate measured
Balasegaram 2006	[67]	Human	TBG in DRC	Melarsoprol and eflornithine	In late HAT, more patients died with melarsoprol alone than eflornithine alone.
Pepin 2000	[68]	Human	TBG in DRC	Eflornithine given to relapsing patients	7-day treatment reduced relapse
WHO 2001	[69]	Human	HAT	HATr	New drugs including DFMO, DB, trypanothione inhibitors, antagonists of polyamine metabolism, nitroimidazoles, combination therapy

### 3.3. Evidence of Human African Trypanocide Resistance of TbAT1 in Clinical Studies

Here, we provide evidence with a total random effect proportion of 68.0% (95% CI: 38.0–91.6) being reported from 2001–2014 (Table 3). Test for heterogeneity: Q (df) = 99.7 (3), *p* < 0.0001. I^2^ = 96.99% (95% CI: 94.6–98.3). The high I^2^ value indicates great variation, which could be associated with the different study designs, time scope, and geographical locations for these studies. Egger’s test (Intercept = −16.5, 95% CI: −30.8 to −2.1, *p* = 0.04), Begg’s test (Tau = −1.0, *p* = 0.04) showed publication bias. Pati [48] reported a further proportion of 4/6 (*Tb*AT1) having mutations associated with the AQP2/3 genes.

### 3.4. Human African Trypanocide Treatment Relapse Rates

Treatment failure rates were highest with melarsoprol, followed by eflornithine, 41.49% (95% CI: 24.94–59.09) and 6.56% (3.06–11.25), respectively; however, the reliability of these findings may be biased due to the high I^2^ value associated with melarsoprol studies (Table 4). Furthermore, a high level of consistency was associated with nifurtimox studies (I^2^ = 0.0%) since this showed a low publication bias.

Treatment relapse rates have been used as indicators of resistance and combination therapies using DFMO-suramin against arsenical/melarsoprol resistance have been explored since eflornithine alone is ineffective [74]. This has continued as an alternative combination for use in late HAT cases because of melarsoprol resistance [75]. To overcome arsenical resistance, combination therapies of metronidazole and suramin have also been used since these are associated with mild side effects when compared with suramin monotherapy (in Zambia), while high doses of nifurtimox have also been used in DRC. Melarsoprol resistance in DRC has been associated with mutations in the AQP2/3 gene [35] and parasite regrowth[56]. Human samples from Tanzania and Ivory Coast have shown cross resistance to melarsoprol and suramin due to alterations in *Tb*AT1 [30] or other transport mechanisms [15].

Melarsoprol resistance in Uganda and Tanzania has also been associated with mutations in the *Tb*AT1 gene [57]. In DRC, melarsoprol alone is no longer used in late-stage HAT [58] and has been substituted with eflornithine for this stage [59]. The increasing levels of HATr have led to the promotion of combination therapy [60], as well as increased capital to invest in the discovery of more potent drugs superior those currently on the market. Furthermore, melarsoprol toxicity and decreasing efficacy has led to phasing out the drug as a frontline treatment against *T. b. gambiense*; this is now possible with the emergence of potent, safe combination chemotherapies, such as NECT, showing that eflornithine will continue to be around for decades to come. The *Tb*AT1 genotype was the most prevalent marker, although few studies have been conducted in humans on the African continent exploring the genome diversity of HATr. This is important since melarsoprol has the highest treatment failure rates [66,73].

### 3.5. Drug Sensitivity Profiles on HATr Using Resistance Profiling

**Figure 2:**
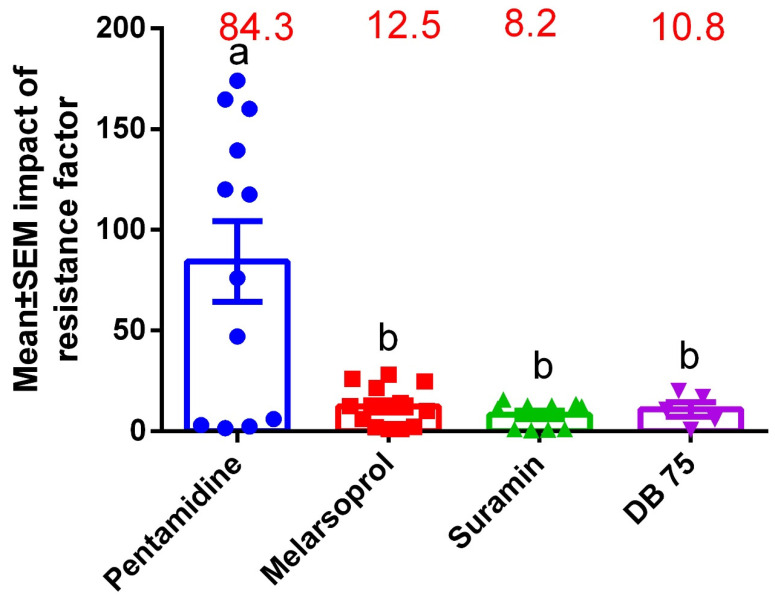
Resistance profiles of laboratory-developed HATr phenotypes. Generally, much emphasis has been placed on development of strains heavily expressing resistance to pentamidine compared with all other trypanocides. Different subperscripts in figure 2 are introduced under statistical analysis (i.e., different superscripts (a,b) signify significant differences).

Some experimental laboratory studies have proved to be unreliable due to low reproducibility, especially when conducting clinical (field-based) studies [76]. This has subsequently given rise to speculative interpretations that make it hard to inform policy [77]. Here, we investigated the level of resistance induced in experimental studies for HATr. The resistance factor was calculated by dividing the IC50 of the resistant population by the IC50 of the wild-type in these studies [13,18,20,29,40]. The mean ± SEM for resistance factors of pentamidine, melarsoprol, suramin, and DB 75 were 84.3 ± 20.12, 12.5 ± 3.0, 8.2 ± 2.0, and 10.8 ± 3.5, respectively (Figure 2). Furthermore, most laboratory studies have continued to produce strains that are significantly resistant to pentamidines, demonstrating a shift in research direction for the next novel trypanocides.

## 4. Conclusions

Cross-resistance across trypanocides is a major threat to the development of novel monotherapy due to the targeting of similar molecules in the pathogen. *Tb*AT1/P2 are the leading pathogenic transporter targets; however, total resistance is associated with the loss of *Tb*AQ2/3, HAPT1, and LAPT1 in melarsoprol-pentamidine resistance, while AAT6 and NTRs are common in nifurtimox–eflornithine resistance. High treatment failure rates in humans have led to the implementation of high doses, which have proved fatal to patients, highlighting the desperate situation created by HATr in endemic communities.

## Figures and Tables

**Table 3:** Proportion of *Tb*AT1 loss in human clinical studies.

Study	Ref	Sample size	Proportion (%)	95% CI	Weight (%)	
					Fixed	Random
Kazibwe 2009	[71]	179	92.7	87.9–96.1	46.63	25.63
Matovu 2001	[66]	68	55.9	43.3–67.9	17.88	25.04
Nerima 2007	[59]	105	89.5	82.0–94.7	27.46	25.37
Pati 2014	[48]	30	20.0	7.7–38.6	8.03	23.95
Total (fixed effects)		382	81.9	77.7–85.6	100	100
Total (random effects)		382	68.0	38.0–91.6	100	100

**Table 4:** Human African trypanocide relapses following pentamidine, nifurtimox, eflornithine, and melarsoprol therapy in humans.

Study	Ref	Pentamidine		Nifurtimox		Eflornithine		Melarsoprol		Combination Melarsoprol/Eflornithine	
		Relapse	Total	Relapse	Total	Relapse	Total	Relapse	Total	Relapse	Total
Balasegaram 2006	[73]	33	692								
Balasegaram 2006	[67]					11	136	36	258		
Brun 2001	[50]							8	36		
Burri 2001	[70]							7	16		
Kagira 2007	[52]							5	6		
Kazibwe 2009	[71]							9	101		
Matovu 2001	[66]							43	65		
Mpia 2002	[55]							19	42	2	42
Pati 2014	[48]							30	45		
Pepin 1989	[63]							12	19		
Pepin 1989	[63]			0	7						
Pepin 1992 *	[64]			0	9			9	30		
Pepin 2000 ^#^ in Côte d’Ivoire	[68]			0	33	0	33				
Pepin 2000 ^#^ in DRC	[68]					7	140				
Pepin 2000 ^#^ in Uganda	[68]					13	116				
Total		33	692	0	49	31	425	178	618	2	42
Total random effects		NA		1.32		6.56		41.49		NA	
95% CI				0.043–6.17		3.057 to 11.252		24.944 to 59.094			
Test for heterogeneity, Q(df), *p* value I^2^ (inconsistency), 95% CI				0.34 (2), *p* = 0.85. I^2^ = 0.00%, 0.00 to 79.99		8.43 (3), 0.038 I^2^ = 64.43%, 0.00–87.95		150.68 (9), *p* < 0.0001; I^2^ = 94.03%, 90.93–96.06			
Publication bias: Egger’s test intercept (95% CI, *p* value); Begg’s test Kendall’s Tau, *p* value				1.036 (0.99–1.08, 0.002); Tau =1.00, *p* = 0.117)		−4.1621 (17.6527 to 9.3285, *p* = 0.3156); Tau = 0.0000, 1.0000		5.7053 (0.5442 to 10.8664), *p* = 0.0342; Tau = 0.2000, *p* = 0.4208			

Superscript: (*) denotes toxic observations; 8 patients developed neurological conditions and 1 died under high nifurtimox dosage. ^#^ Observations taken within a 2-year period. NA = Not applicable for meta-analysis of a low number of studies (*n* = 1).

## Data Availability

All information used in the study is available in the manuscript.

## Supplementary Materials

The following are available online at https://www.mdpi.com/article/10.3390/pathogens11101100/s1, File S1: keyword search criteria on HATr.
