# Supplementary material for: Systematic Review and Meta-Analysis on Human African Trypanocide Resistance
Source: Pathogens. 2022 Sep 25;11(10):1100. doi: 10.3390/pathogens11101100 (PMC9612373; doi:10.3390/pathogens11101100)
Supplement: Supplementary file 1 [file pathogens-11-01100-s001.zip › pathogens-1856665-supplementary.pdf]

Supplementary file S1: Keyword Database search criteria

AMED (Allied and Complementary Medicine) <1985 to March 2022>

CAB Abstracts <1973 to 2022 Week 13>

APA PsycInfo <1806 to March Week 4 2022>

Books@Ovid <March 28, 2022>

Journals@Ovid Full Text <April 01, 2022>

Your Journals@Ovid

APA PsycArticles Full Text

CAB Abstracts <1910 to 1989>

Embase Classic+Embase <1947 to 2022 April 01>

Global Health <1910 to 2022 Week 13>

Ovid MEDLINE(R) and Epub Ahead of Print, In-Process, In-Data-Review & Other Non-Indexed Citations, Daily and Versions <1946 to April 01, 2022>

1 exp African trypanosomiasis/ 20006

2 african trypanosomiasis/ or trypanosoma brucei/ or trypanosomiasis/ or tsetsefly-borne diseases/ 97596

3 exp trypanosoma gambiense/ 13678

4 exp trypanosoma rhodesiense/ 13672

5 exp trypanocides/ 40634

6 exp suramin/ 12879

7 exp melarsoprol/ 2171

8 exp eflornithine/ 7707

9 exp nifurtimox/ 4300

10 Trypanosoma gambiense.od. 6512

11 exp pentamidine/ 14248

12 Trypanosoma.od. and eflornithine.sh. and Trypanosoma gambiense.od. and nifurtimox.sh. 24

13 exp drug resistance/ 937256

14 1 and 220006

15 HAT.mp. [mp=ab, hw, ti, ot, bt, id, cc, tc, tm, mf, tx, ct, tn, dm, dv, kf, fx, dq, nm, ox, px, rx, ui, sy] 63632

16 14 or 15 81076

17 3 or 4 17702

18 16 and 17 10047

19 6 or 7 or 8 or 9 or 11 or 12 37790

20 exp pentamidine/ 14248

21 19 or 20 37790

22 18 and 21 1251

23 13 and 22 172

Breakdown per database:

13 and 22	172
CAB Abstracts <1973 to 2022 Week 13>	45
CAB Abstracts <1910 to 1989>	5
Embase Classic+Embase <1947 to 2022 April 01>	1
Global Health <1910 to 2022 Week 13>	93
Ovid MEDLINE(R) and Epub Ahead of Print, In-Process, In-Data-Review & Other Non-Indexed Citations, Daily and Versions <1946 to April 01, 2022>	28

URL: https://www.ezproxy.is.ed.ac.uk/login?url=http://ovidsp.ovid.com/ovidweb.cgi?T=JS&NEWS=N&PAGE=main&SHAREDSEARCHID=4rS3nFG7ZyFYFrGaPC86rkWf4dznjeUBLFzxmyVWoBq7avEPXOhTNZDrlgM0v4HVM

Web of Science Interface

(((((ALL=((african trypanosomiasis)) OR ALL=(trypanosoma brucei)) OR ALL=('tsetsefly-borne diseases' OR 'HAT' or ‘human African trypanosomiasis’))) AND ALL=((trypanosoma brucei gambiense) or (trypanosoma rhodesiense))) AND ALL=((suramin OR melarsoprol OR eflornithine OR nifurtimox OR pentamidine OR (NECT or (Nifurtimox Eflornithine Combination Therapy))))) AND ALL=(trypanocides resistance or drug resistance) 110 papers

URL: https://www.webofscience.com/wos/woscc/summary/4176f201-9404-43a1-967c-515eb4e2b1d8-2e9a627f/relevance/1
